# Enhancing practitioners’ confidence in recruitment and consent in the EcLiPSE trial: a mixed-method evaluation of site training – a Paediatric Emergency Research in the United Kingdom and Ireland (PERUKI) study

**DOI:** 10.1186/s13063-019-3273-z

**Published:** 2019-03-21

**Authors:** Kerry Woolfall, Louise Roper, Amy Humphreys, Mark D. Lyttle, Shrouk Messahel, Elizabeth Lee, Joanne Noblet, Anand Iyer, Carrol Gamble, Helen Hickey, Naomi Rainford, Richard Appleton

**Affiliations:** 1 Department of Health Service Research, Institute of Population Health and Society, University of Liverpool, Liverpool, UK; 2 Clinical Trials Research Centre (CTRC) North West Hub for Trials Methodology, University of Liverpool, Liverpool, UK; 3 Emergency Department, Bristol Royal Hospital for Children, Bristol, UK; 4 Faculty of Health and Applied Sciences, University of the West of England, Bristol, UK; 5 Emergency Department, Alder Hey Children’s NHS Foundation Trust, Liverpool, UK; 6 Neurology Department Alder Hey Children’s NHS Foundation Trust, Liverpool, UK

**Keywords:** Clinical trials, Practitioner training, Research without prior consent, Deferred consent, Paediatric emergency care, Recruitment

## Abstract

**Background:**

EcLiPSE (Emergency treatment with Levetiracetam or Phenytoin in Status Epilepticus in children) is a randomised controlled trial (RCT) in the United Kingdom. Challenges to success include the need to immediately administer an intervention without informed consent and changes in staffing during trial conduct, mainly due to physician rotations. Using literature on parents’ perspectives and research without prior consent (RWPC) guidance, we developed an interactive training package (including videos, simulation and question and answer sessions) and evaluated its dissemination and impact upon on practitioners’ confidence in recruitment and consent.

**Methods:**

Questionnaires were administered before and immediately after training followed by telephone interviews (mean 11 months later), focus groups (mean 14 months later) and an online questionnaire (8 months before trial closure).

**Results:**

One hundred and twenty-five practitioners from 26/30 (87%) participating hospitals completed a questionnaire before and after training. We conducted 10 interviews and six focus groups (comprising 36 practitioners); 199 practitioners working in all recruiting hospitals completed the online questionnaire. Before training, practitioners were concerned about recruitment and consent. Confidence increased after training for explaining (all scale 0–5, 95% CIs above 0 and *p* values < 0.05): the study (66% improved mean score before 3.28 and after 4.52), randomisation (47% improvement, 3.86 to 4.63), RWPC (72% improvement, 2.98 to 4.39), and addressing parents’ objections to randomisation (51% improvement, 3.37 to 4.25). Practitioners rated highly the content and clarity of the training, which was successfully disseminated. Some concerns about staff availability for training and consent discussions remained.

**Conclusions:**

Training improved practitioners’ confidence in recruitment and RWPC. Our findings highlight the value of using parents’ perspectives to inform training and to engage practitioners in trials that are at high risk of being too challenging to conduct.

**Electronic supplementary material:**

The online version of this article (10.1186/s13063-019-3273-z) contains supplementary material, which is available to authorized users.

## Background

Many clinical trials experience difficulties in recruiting the desired number of participants, resulting in underpowered studies and continued use of healthcare treatments that are not informed by scientific evidence [1–4]. Trials involving time-critical interventions in paediatric emergency and critical care have additional practical and ethical challenges. These include, high staff turnover due to junior physician rotations, no time to seek prior informed consent, and practitioner anxiety about approaching families for consent after a trial intervention has already been administered [5].

Emergency Treatment with Levetiracetam or Phenytoin in Status Epilepticus in Children (EcLiPSE) was a 30-site, un-blinded, pragmatic and randomised controlled trial that explored second-line treatment (levetiracetam versus phenytoin) of convulsive status epilepticus in children [6]. EcLiPSE was one of the first UK paediatric clinical trials of an investigational medical product (CTIMP) to randomise and treat patients without seeking prior informed consent from parents. As there is no time to seek written informed consent in a life-threatening situation, practitioners approached parents as soon as possible after the child has stabilised to inform them that their child has already been entered into a clinical trial and discuss the use of their child’s data and continued follow-up [7, 8]. This is research without prior consent (RWPC, also known as deferred consent). Challenges to the success of EcLiPSE included: practitioner concerns and inexperience in RWPC; trained staff leaving due to junior physician rotations; use of an anti-epileptic medication (levetiracetam) that is not the standard anticonvulsant used in this clinical setting; and the likelihood that randomised patients are moved between departments or hospitals, which increases the size of trial team and complexity of trial set up.

The CONseNt methods in paediatric Emergency and urgent Care Trials (CONNECT) study [7, 9] explored parent and practitioner perceptions and experiences of RWPC in paediatric and neonatal trials. CONNECT found that practitioners with no experience of RWPC might have negative perceptions of this consent process [10]. Parents and experienced practitioners who participated in CONNECT indicated support for this approach to consent, although some parents raised concerns about trials which involved interventions not commonly used in routine clinical practice. CONNECT interviews also explored parents’ perspectives on the EcLiPSE trial design, including approach to recruitment, consent, and patient information materials [11]. Parents made specific recommendations on potential approaches to recruitment and consent in EcLiPSE. This included the need to appropriately time the EcLiPSE consent discussions and to discuss the safety of the trial interventions, as well as how both EcLiPSE interventions are used in routine clinical practice [11].

A recent systematic review highlighted the need to develop practitioner training to improve recruitment and consent in trials [12]. Site initiation visits (SIVs) are used to engage practitioners with the study aims and procedures and how to deliver the clinical trial protocol [5]. In the early design stage, we recognised the need for a comprehensive training package to educate and help provide practitioners with confidence in recruitment and consent. The SIV training package included specific training on RWPC and recommendations made by parents to inform approaches to recruitment and consent seeking in EcLiPSE using CONNECT study findings and associated guidance [7, 9, 11, 13]. In this embedded study (called the Consent study) we evaluated the effectiveness of the EcLiPSE SIV training on practitioners’ confidence in recruitment and consent seeking as well as the effectiveness of training dissemination.

## Methods

### Study design and setting

We chose a mixed-method longitudinal design [14], to provide us with different forms of data and insights from multiple practitioner perspectives throughout the trial as part of an iterative process [15–17]. We designed a semi-structured questionnaire (see Additional file 1) administered immediately before (Part A) and after (Part B) the SIV training. This included four statements aimed to assess practitioners’ confidence in aspects of recruitment and consent with five response options (‘strongly disagree’ to ‘strongly agree’). We then conducted telephone interviews with recruitment and training leads at the first sites open and conducted focus groups drawn from high and low recruiting sites at the end of the first year of recruitment with a mix of nurses, research nurses and consultants. We also sent an online questionnaire to all sites in last phase of the trial (approximately 8 months before trial closure). The online questionnaire was sent to lead practitioners and research nurses at the study sites and they were asked to distribute it to staff at their site who were trained in EcLiPSE. Topic guides (see Additional file 2) and the online questionnaire were designed to explore recruitment, RWPC experience, barriers to training and any problems and potential solutions to trial conduct.

### Selection of participating sites

EcLiPSE sites were part of the Paediatric Emergency Research in the United Kingdom and Ireland (PERUKI), a paediatric emergency medicine (PEM) research collaborative, which includes tertiary and district general hospitals, with varying levels of research experience [5]. In 26 of the 30 EcLiPSE sites KW, LR or AH provided a brief description of the evaluation before the opening presentation (see Table 1) and invited practitioners who intended to stay for the full training to participate in the evaluation by completing Part A of the questionnaire before and Part B at the end of training. The need for the SIV evaluation was identified after the first SIV. Three other sites were not included due to either their participation in a different training evaluation study (*n* = 2) or full SIV training adapted due to low attendance (*n* = 1). Questionnaire completion was taken as indication of consent. Personal details were not requested to ensure anonymity.

**Table 1:** Site training content and delivery

Section number and content	Content (recruitment and consent related content in italic text)	Aim/key message (recruitment- and consent-related content in bold text)	Method of delivery (interactive element in bold text)	Typical length (mins)	Who delivered the session?
1. Site initiation evaluation questionnaire	Description of site evaluation aims How and when to complete Part A and Part B Invitation to complete Part A before the opening presentation	Methods for site initiation evaluation To highlight aims, anonymity of participation and implied consent To provide an opportunity for questions	One-to-one or small group discussion as practitioners arrived	5	Consent study team or trial co-ordinator
2. Opening presentation	Rationale and trial aims and *approach to consent* Introduction to the trial team	To highlight the significance of the trial, key aspects including trial interventions and *approach to consent* Encourage practitioner engagement	PowerPoint presentation	15–20	Trial co-ordinator/ consultant/research nurse
3. Protocol overview including sample handling	Study endpoints, inclusion and exclusion criteria Protocol for staff Sample processing procedures	To outline key aspects of the protocol to ensure that it was followed in full including *follow-up outside of the ED for consent procedures*	PowerPoint presentation, *question and answer session*	15–20	Trial co-ordinator
4. ED essentials	Screening and randomisation Treatment administration and accountability Case Report Form (CRF) completion *Brief overview of approach to recruitment and consent (RWPC)*	*To consider the patient pathway, randomisation and intervention administration in the ED* *To provide practitioners with guidance on addressing parents’ trial-related questions during resuscitation*	PowerPoint Presentation, *video* (covering screening, randomisation and intervention administration) and *real-time simulation* of screening, randomisation and intervention administration and *question and answer session*	60	Trial co-ordinator/ consultant/research nurse
5. Research without prior consent (deferred consent)	*Pre-trial research findings and related research/guidance* [7, 11] *Approach to recruitment and consent in EcLiPSE* *.Overview of forms including Patient information sheets and consent forms*	*To provide evidence that describes how parents support the trial and research without prior consent* *To guide practitioners on when to approach and discuss RWPC with parents, including what should happen in the situation when a child has died after entry into the trial*	PowerPoint presentation – *RWPC scenario video base on pre-trial research findings* *Question and answer session specific to recruitment and consent*	60–90	Consent study team member or trial co-ordinator
6. Safety reporting and monitoring	Definitions of adverse events etc. and process of reporting (including forms to use) Guidance for CRF completion	To guide practitioners on how to complete AE forms and report AEs To provide practitioners with data protection knowledge, monitoring visits information, PI obligations and document storage and archiving knowledge	PowerPoint presentation, *question and answer session*	15	Trial co-ordinator
7. Next steps and question and answer	Outline of trial progress and expected site opening Description of how training should be rolled out to all staff on a regular basis	Opportunity for questions on any issue including rolling out of training	*Group discussion*	15–20	Led by trial co-ordinator, questions answered by all team members present
8. Completion of Part B site evaluation questionnaire	Reminder to complete Part B before leaving the session	Completion of site evaluation questionnaire (Part B)	Invitation to complete questionnaire and leave for collection before leaving the room	5	Consent study team or trial co-ordinator

*AE* adverse event, *ED* emergency department, *CRF* Case Report Form, *RWPC* research without prior consent

LR (female health psychologist, CPsychol) emailed lead practitioners at 16 high (exceeding recruitment target) and low (below recruitment target) recruiting sites inviting them and their colleagues to participate in a focus group at their site. Potential participants were not known to the qualitative team who had no prior experience of SIV training. Telephone interviews were conducted with lead practitioners at sites open in the first year of trial recruitment. Practitioners were eligible if they been involved in the recruitment and/or consent of at least two trial participants. Consent was sought for interview and focus group participation, including consent for audio recording. The trial co-ordinator (AH) emailed sites 8 months before the scheduled trial end date and invited staff to complete the online questionnaire. ML sent email reminders on behalf of the trial team and PERUKI. It was anticipated that some of the same staff who took part in a telephone interview or focus groups would also complete the online questionnaire.

### Training package

We developed the recruitment and consent training, including a RWPC-scenario video, using CONNECT study guidance on RWPC, pre-trial feasibility work involving parents [7, 11] and Clinical Trials Research Centre (Clinical Trials Unit) Standard Operating Procedure guidance [18]. Printed materials were provided which included: guidance on RWPC [7]; published trial feasibility findings [11] and trial materials, such as participant information sheets, screening form and infusion guidelines (available on request). To assist ongoing training dissemination for new staff (e.g. after junior physician rotations) all materials were given to the site lead practitioner on a USB stick and made available to all practitioners via the study website.

Site training included presentation of protocol, screening and randomisation simulation (video and real-time), RWPC (presentation and consent discussion scenario video [https://www.liverpool.ac.uk/psychology-health-and-society/research/connect/resources/]), safety and reporting and question and answer sessions (see Table 1). Emergency department (ED) staff not expecting to be involved in recruitment discussions with families were given a brief overview of the approach to recruitment and RWPC in EcLiPSE (see Section 4, Table 1) and provided with the option of staying for the full RWPC training (see Section 5, Table 1).

Two to five (mean 3.2, mode 3) EcLiPSE team members delivered the EcLiPSE training; typically, the trial co-ordinator, a consultant-level physician (in PEM, paediatric neurology, or chief investigator) or research nurse and a member of the embedded study team (social scientist or health psychologist). Site training lasted for approximately 4 h and took place in a hospital meeting room.

### Analysis

Questionnaire data were entered into SPSS. Descriptive statistics are presented with percentages and the chi-square test for trend, paired-samples *t* test and Wilcoxon signed-rank test (confidence interval 95%) used as appropriate. Questionnaires with recruitment and consent-related data missing were excluded from analysis. To investigate the presence of informative missing data, the results of those who completed only the Part A (before training) questionnaire were compared with those who completed the Part A and Part B (after training) questionnaire. This was also undertaken for those who completed only the ‘after’ questionnaire. LR and KW used NVivo 10 Software to assist in the organisation and coding of interview and focus group data and free-text questionnaire responses. Qualitative thematic analysis [17] was broadly interpretive and iterative [19, 20]. Interviews were conducted until a representative from each site open in the first year of recruitment had been interviewed. Focus groups were conducted until data saturation (no new major themes were discovered in analysis [17, 21]). Our approach to synthesising qualitative and quantitative data [22] drew on the constant comparative method [23, 24].

## Results

### Characteristics of the study subjects

A total of 333 practitioners received EcLiPSE training by attending SIVs. An average of 11 staff attended each SIV (range 3–18). Three hundred and twelve (94%) practitioners from 26 of the 30 (87%) sites anticipated being involved in consent processes. One hundred and forty-nine of 312 (48%) practitioners were eligible for inclusion as they anticipated staying for the full training session. Clinical commitments impacted attendees’ ability to attend the entire SIV; consequently, 24/149 (16%) were partially completed and excluded from analysis due to missing data. Of the 125 (45 nurses, 57 physicians, 23 other (e.g. pharmacist)) participants, many (84/125, 67%) had previous experience of conducting clinical trials (range 1–300 months, mean 54 months experience), whilst 24 (19%) had experience of RWPC in trials.

**Fig. 1:**
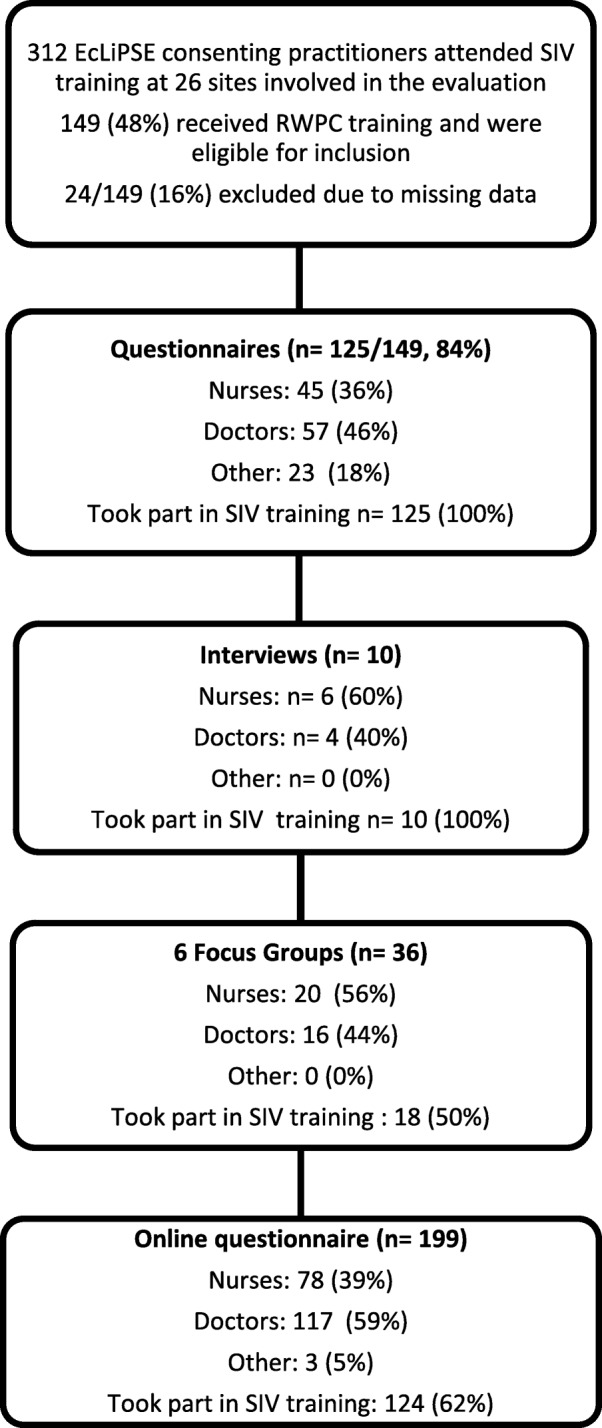
Study methods and participant characteristics. Figures are *n* (%). Abbreviation: *SIV* site initiation visit

As shown in Fig. 1, LR telephone interviewed principal investigators (PIs) (*n* = 4) or lead research nurses (*n* = 6) and conducted six focus groups at six hospitals with 36 practitioners (20 nurses and 16 physicians, including three PIs). Focus groups took on average 60 min and interviews 40 min. Only the researcher and participants were present. Telephone interviews took place 8–18 months (mea*n* = 11 months and 10 days, range 260–577 days) post SIV training and 5–16 months (mean 8 months and 21 days, range 168–490 days) after site opening. All (10/10, 100%) telephone interview participants had attended the SIV training. None of the PIs or research nurses had prior experience of RWPC in paediatric trials; one physician had comparable experience in an adult trial. Focus groups were held 13–18 months post SIV (mean = 14 months and 29 days, range 400–574 days) and mean 12 months and 9 days (range 329–420) after site opening. Half (18/36, 50%) of focus group participants had attended SIV training. A total of 199 practitioners from all 28 recruiting hospitals completed the online questionnaire 8 months before the end of the trial. Of these, 124/199, (62%) had attended SIV training. The other practitioners had attended subsequent EcLiPSE training sessions facilitated by their site.

## Results

### Practitioner concerns about recruitment and consent in EcLiPSE before site training

Thirty-three of 118 practitioners (28%) who completed a questionnaire cited concerns about recruiting patients to EcLiPSE; a slightly higher proportion (48/120, 40%) were concerned about seeking consent (RWPC) (see Table 2). Previous experience of RWPC was not associated with concerns about recruitment or seeking consent in EcLiPSE.

**Table 2:** Concerns about recruitment and consent in EcLiPSE by practitioner experience of research without prior consent (RWPC) (*n* = 125)

Question	Yes *n* (%)	No *n* (%)	Odds ratio	95%CI	*p* value
1. Do you have any concerns about recruiting to EcLiPSE?	33 (28)	85 (72)			
*Experienced in RWPC*	*4 (19)*	*17 (71)*	*0.54*	*1.17–1.74*	*0.296*
*Not experienced in RWPC*	*28 (30)*	*64 (70)*			
2. Do you have any concerns about seeking consent for EcLiPSE?	48 (40)	72 (60)			
*Experienced in RWPC*	*6 (26)*	*17 (74)*	*0.46*	*0.17–1.30*	*0.128*
*Not experienced in RWPC*	*40 (43)*	*52 (57)*			

Figures are *n* (%), percentages are rounded to the nearest whole number. Missing responses: question 1, *n* = 7, 5.6%; question 2, *n* = 5, 4.0%. Missing responses for cross tabulation by experience of RWPC: question 1, *n* = 12, 9.6%; question 2; *n* = 10, 8.0%

To explore this further, the questionnaire included four additional statements to specifically assess practitioners’ confidence in aspects of recruitment and RWPC in EcLiPSE before site training. The results grouped by experience of RWPC for statements 1 to 4 are presented in Table 3.

**Table 3:** Practitioner confidence in aspects of consent seeking by experience of research without prior consent (RWPC) before and after training (*n* = 125)

I feel confident in…..	Before training						After training						Changes in confidence after training				
	Strongly disagree	Mildly disagree	Neither agree nor disagree (neutral)	Mildly agree	Strongly agree	Mean confidence score	Strongly disagree	Mildly disagree	Neither agree nor disagree (neutral)	Mildly agree	Strongly agree	Mean confidence score	Improved	Decreased	No change	Mean difference (95% CI)	*p*-value
1.Explaining the study to families	11 (9)	27 (22)	30 (24)	30 (24)	27 (22)	3.28	0 (0)	0 (0)	4 (3)	52 (42)	69 (55)	4.52	82 (66)	4 (3)	39 (31)	− 1.24 (− 1.46, − 1.02)	0.000^a^
2.Explaining randomisation to families	8 (6)	7 (6)	20 (16)	49 (39)	41 (33)	3.86	0 (0)	0 (0)	2 (2)	42 (34)	81 (65)	4.63	58 (47)	3 (2)	64 (51)	− 0.77 (− 0.96, − 0.57)	0.002^b^
3.Explaining RWPC to families	20 (16)	24 (19)	40 (32)	21 (17)	20 (16)	2.98	0 (0)	1 (1)	12 (10)	48 (39)	61 (50)	4.39	90 (72)	2 (2)	32 (26)	− 1.40 (− 1.62, − 1.17)	0.000^a^
4. Dealing with parents who object to their child being randomised	8 (6)	28 (22)	29 (23)	30 (24)	30 (24)	3.37	0 (0)	2 (2)	16 (13)	56 (45)	51 (41)	4.25	64 (51)	5 (4)	56 (45)	− 0.88 (− 1.08, − 0.68)	0.000^b^

^a^paired-sample *t* test, ^b^Wilcoxon signed-rank test

Figures are *n* (%). Totals may not equal 100% due to rounding

Missing responses: statement 3 *n* = 3, 2% (after training)

Just under half (57/125, 46%) responded positively to statement 1, indicating confidence in explaining the study to families. The majority (90/125, 72%) indicated confidence in explaining randomisation to families (statement 2). Previous experience of RWPC did not significantly improve levels of confidence in explaining either the study (*p* = 0.88), or the process of randomisation to families (*p* = 0.49).

Of the four statements, practitioners indicated they were least confident in explaining RWPC to families (statement 3). Thirty-three percent (41/125) responded positively. Although only two practitioners with previous experience of RWPC indicated a lack of confidence in the method, compared to 41% (39/69) of practitioners without experience of RWPC, there appeared to be some uncertainly about RWPC even amongst those with prior experience. Just under half of those with experience of RWPC (10/24, 42%) responded in the ‘neither agree not disagree’ option to statement 3.

In response to statement 4, just under half (60/125, 48%) of practitioners responded positively, indicating that they felt confident in dealing with parents who might object to their child being randomised. Prior experience of RWPC was also not associated with increased confidence in dealing with parents’ objections at the point of randomisation (*p* = 0.288). Themes identified in the analysis of interview and focus group data as well as qualitative free-text responses supported quantitative questionnaire findings. As shown in Table 4, many practitioners described how their concern arose from inexperience and lack of knowledge about RWPC. Some were ‘*quite concerned how parents would take that* (RWPC)’ (P1, focus group 4, female, nurse and about ‘*How to approach it with the parents*’ (P3, interview, female, nurse) after their child had been entered into EcLiPSE.

**Table 4:** Main themes related to practitioner concerns about recruitment, consent and the practicalities or logistics of conducting EcLiPSE before and after training identified in qualitative questionnaire free-text responses (*n* = 125), interviews (*n* = 10) and 6 focus groups (*n* = 36)

Concerns before training		Concerns after training	
Recruitment and consent	Practical or logistical	Recruitment and consent	Practical or logistical
Main themes identified in *n* = 62 free-text responses to Part A question: *Do you have any concerns about seeking RWPC/deferred consent?* and *n* = 48 and Part A question: *Do you have any concerns about recruiting to EcLiPSE?* and in *n* = 9/10 interviews and in *n* = 3/6 focus groups	Main themes identified in *n* = 95 open-ended responses to questionnaire Part A question: *Do you think there will be any practical or logistical difficulties with conducting EcLiPSE?* and *n* = 9/10 interviews and in *n* = 3/6 focus groups	Main themes identified in *n* = 32 free-text responses to Part B question: *Do you have any concerns or anxieties specific to this trial including the consent approach we have discussed?* and *n* = 5/10 interviews and in *n* = 1/6 focus groups	Main themes identified in *n* = 32 free-text responses to Part B question: *Do you think there will be any practical or logistical difficulties with conducting EcLiPSE?* and *n* = 4/10 interviews and in *n* = 1/6 focus groups
Lack of knowledge about the trial *‘I do not know enough about the study to confidently recruit.’* (P9, SIV questionnaire, female ED nurse) *‘Get our heads round the purpose of the study.*’ P8, interview, female, nurse *‘Lack of familiarity*.’ (P3, focus group 1, male, physician)	Staffing *‘Deferred consent over the weekend*.*’* (P55, SIV questionnaire Part A, female, physician) *‘At the time it was only going to be myself and […], my research nurse, and I knew that that could put a lot of pressure on us if we had to be available pretty much 365 days a year.’* (P1, interview, male, physician)	Seems feasible but experience needed *‘I guess we will have to give it a go.’* (P7, SIV questionnaire Part B, female, nurse) *‘Just my own inexperience of using the deferred consent approach however it seems much more feasible now I have completed the training.’* (P128, questionnaire Part B, female, physician) *‘My experience of it is that I need more experience of it. Because I feel a bit cautious.’* (P3, focus group 1, female, physician)	Staff availability for consent *‘Logistics of ensuring 7-day provision of consenting team members.’* (P21, SIV questionnaire Part B, male, physician) *‘We did miss a patient because he (trained consenting staff member) wasn’t around, and it was over a weekend and we don’t work at the weekend, so there has been an eligible patient.’* (P1, telephone interview, female, nurse). *‘People are trained and people are happy to do it, but we haven’t yet tested it when there‘s potentially one person who is able to do it, but the shop floor is crazy. We’ll see what happens over the winter with that.’* (P1, focus group 1, male, physician)
Lack of experience in RWPC *‘I feel nervous about deferred consent issue purely because this is new for me.’* (P50, SIV questionnaire Part A, female, nurse) *‘I guess we had initial reservations about the deferred consent and how that would work because it was something that was very much new to us’* (P4, interview, female, physician) *‘I’d never used deferred consent before. So it’s the first study that we’re using it.’* (P1, focus group 4, female, nurse)	Staff awareness *‘Familiarity of ED staff with process’* (P3, SIV questionnaire Part A, female, nurse) *Doctors may forget it if not trained and aware* (P7, SIV questionnaire, female, nurse) *‘The big concern I had was just making sure that everyone was, I suppose, on board with the process. So remembering that the EcLiPSE is ongoing… and if you do remember that the EcLiPSE study is on, you know what to do when they come in.’* (P7, interview, male, physician)	Parents‘ responses to RWPC *‘Negative reaction from parents.’* (P27, SIV questionnaire Part B, female, nurse) *‘It’s brilliant looking at videos and all the rest of it but you never actually know what’s going to happen until you’re in that situation yourself. I think most of us don’t have kids, we’re all a bit nervous about approaching the parents. You’re like, oh my God, what are they going to say, what are they going to do?’* (P6, interview, female, nurse) *‘I didn’t think many parents would be too impressed with it (RWPC), personally.’* (P6, focus group 5, female, nurse)	Dissemination of training *‘Need to create the same awareness as an accepted means of research methodology’* (P124, SIV questionnaire Part B, female, physician) *‘I realised that to try and let the whole children’s hospital know that we’re doing this study is actually quite a challenge in itself.’* (P5, interview, male, physician). *‘Then we realised that as the doctors change, we’ve also then got to remind everyone to resend the email to hopefully get it cascaded round the rest of the medical team. So you have to keep doing that and try and keep everyone aware.’* (P3, focus group 6, male, nurse)
Lack of knowledge about RWPC *‘But I think probably our main, not that it was a concern, I guess more thoughts, was how would the deferred consent work really.’* (P4, interview, female, physician) *‘I had a lot of concerns. I really thought it wasn’t going to work.’* (P1, focus group 2, female, nurse)	Dissemination of training *‘Training a large amount of staff.’* (P100, SIV questionnaire Part A, female, nurse)		
How to explain RWPC to parents *‘How to word it to parents may be the bigger issue.’* (P91, SIV questionnaire Part A, female, physician) *‘How to approach it with the parents, like some one-liners’* (P3, interview, female, nurse) *‘I didn’t know if I was actually going to be able to converse with them, if I was going to be articulate enough. I was so terrified I just thought I was going to mess it up and nobody was ever going to say yes to me.’* (P3, focus group 5, female, nurse)	Research in a resuscitation situation *‘In acute situation therefore need to prioritise clinical care.’* (P45, questionnaire Part A, female, physician) *‘I thought, oh God, this sounds horrendous. Literally I was thinking, how is this going to work? This is going to be a nightmare.’* (P3, focus group 3, female, physician)		
How parents would respond to RWPC *‘Concerned if parents angry that not asked consent prior to getting drug.’* (P27, SIV questionnaire Part A, female, nurse) *‘I was concerned that that might lead to some potentially conflicting, slightly awkward conversations.’* (P1, interview, male, physician) *‘I was a bit dubious about it, yes, because I’d never experienced deferred consent and I’d not been in research that long so it was a bit... I was of the thought that people might be greeted by a punch in the face or a similar sort of verbal aggression.’* (P3, focus group 6, male, nurse)	Buy-in – getting all staff on board *‘Not full time shop floor job role. Difficult to control for other people who may be less interested in our department being involved.’* (P121, SIV questionnaire Part A, female, physician) *‘It (the problem) wasn’t from the point of deferred consent, it was more from the fact that we have got to try and get all the A&E team on board with a research project.’* (P7, interview, male, nurse) *‘So that was my main concern before the SIV, was just, who else would be willing to do the deferred consent? I’ve got some trainees who would be excellent at it but they’re only going to be around for 6 months and then you’d have to keep training people, so it was making sure that the permanent members of our team would be on board*.*’* (P2, interview, male, physician)		

*SIV* site initiation visit

### Logistical concerns about EcLiPSE before training

Over half (69/115, 60%) of practitioners anticipated that there would be practical or logistical difficulties in conducting EcLiPSE.

For many, EcLiPSE was their first ED-led paediatric clinical trial, which appeared to underpin many of the issues described by practitioners in free-text questionnaire responses (see Table 4). Practitioners were concerned about having adequate research support to conduct consent discussions with families, particularly at weekends, as well as the challenge of training all relevant staff across departments. Others discussed their concerns about following a trial protocol in an emergency resuscitation situation, whilst ensuring the clinical care of critically ill children was not compromised. As the number of eligible patients per site was expected to be low, practitioners referred to the anticipated challenge of maintaining trial awareness to ensure eligible patients were not missed. Principal investigators were most concerned about the importance of engaging and motivating all staff in EcLiPSE to maximise trial success.

### Improved confidence in recruitment and consent after training

As shown in Table 3, improved levels of confidence were observed for all four statements regardless of whether practitioners had prior experience of RWPC. It was notable that following training, 82 (66%, 95% confidence intervals (CIs) 3.28 to 4.52) of practitioners felt their confidence in explaining the study to families was improved, whilst 90 (72%, CI 2.98 to 4.39) felt more confident in explaining RWPC to families. Approximately half of practitioners also indicated their confidence in explaining randomisation (47%, CI 3.86 to 4.63) and addressing parents’ objections to randomisation (51%, CI 3.37 to 4.25) had improved.

Questionnaire Part B (after training) free-text responses, as well as interview and focus group discussions also indicated that EcLiPSE training had addressed many of the practitioners’ concerns about recruitment and RWPC. After training, many described how the trial and its approach to consent seemed more ‘*feasible*’ (P128, SIV questionnaire, female, physician), ‘*logical and straightforward*’ (P30, SIV questionnaire, female, nurse). However, as shown in Table 4, themes identified in free-text questionnaire comments indicated that some practitioners remained anxious that parents would respond negatively to the RWPC discussion. Some practitioners involved in the follow-up interviews and focus groups reflected upon their post-training anxiety about RWPC before their site had opened to EcLiPSE recruitment. They described how despite finding the training videos useful, practitioners may need first-hand experience of RWPC to fully address anxieties about how parents will react to RWPC. Indeed they all reflected on how their EcLiPSE recruitment experience had addressed such anxieties as ‘*It’s all been a really positive response (to RWPC). Like I say, we haven’t had one refusal yet*’ (P8, interview, female, nurse). Some described how, when disseminating EcLiPSE training, they had highlighted the involvement of patients in the trial design and training package to help address concerns about the consent process:

‘*It’s quite powerful to be able to say that parents have been involved in the process of approving deferred consent*’ (P7, interview, male, nurse).

### Valued aspects of the SIV

As shown in Table 5, practitioners rated highly the content and clarity of all sections of the training.

**Table 5:** Content and clarity of training (*n* = 125)

Training section	Content (mean)	Clarity (mean)
a. Introduction to EcLiPSE	4.59	4.62
b. Protocol overview	4.62	4.59
c. Research without prior consent (deferred consent)	4.57	4.55
d. Safety reporting and monitoring	4.56	4.58
e. The emergency department (ED) essentials	4.62	4.59

Figures are means rounded to two decimal places. Missing ‘content‘: a. *n* = 7; b. *n* = 5; c. *n* = 7; d. *n* = 11; e. *n* = 11. Missing ‘clarity‘: a. *n* = 14; b. *n* = 14; c. *n* = 15; d. *n* = 19; e. *n* = 19

Practitioners were asked to rate two statements on a scale of 1 (strongly disagree) to 5 (strongly agree) to indicate whether the training videos had improved their confidence in (1) identifying eligible children and (2) seeking consent; they received mean ratings of 4.28 and 4.31, respectively.

In free-text responses, interviews and focus groups, many described how videos were useful to help visualise processes, including screening, randomisation and seeing a RWPC discussion ‘*in action rather than theory*’ (P21, SIV questionnaire, male, physician). Research nurses in particular valued the examples of tailored communication such as the ‘*terminology used to explain this to families*’ (P109, SIV questionnaire, female, nurse), and ‘*there are some good sort of one-line quotes that you can take from it*’ (P4, interview, female, nurse), as well as ‘*see how nurse handled difficult questions*’ from parents (P27, SIV questionnaire, female, nurse). Practitioners responsible for site training described how the videos and training slides provided had assisted learning and dissemination: ‘*actually what really helped me is then when I was having to give the training to everybody else, I already had the package and I already had the PowerPoint, so that in actual fact helped me reinforce my own learning*’ (P3, interview, female, physician).

During interviews, practitioners spoke of how the involvement of a number of the EcLiPSE team members, including the chief investigator, in delivering the SIV training had helped to create a sense of study importance, which appeared to help engage practitioners:

‘*I think people were very impressed that the chief investigator had arrived… they saw that as a really, really good sign that people were taking this very seriously*’(P1, interview 1, male, physician).

### Logistical concerns after training and support

Although there were fewer logistical concerns described after training (Table 4), some practitioners re-stated concerns about staff availability to cover consent discussions with families 7 days a week. Questionnaire, interview and focus group participants described the anticipated challenge of successfully disseminating training to relevant staff including new physicians at the trainee rotational changeover, particularly over the busy winter period.

Practitioners suggested that the trial management team could provide recruitment and consent support through study updates, advice when required and recruitment-training tips from the ongoing embedded Consent study. This support was provided through regular contact and newsletter updates, which included recruitment tips in addition access to training materials online and on a USB provided to each site PI. Practitioners described how trial team support and access to training materials help facilitate the dissemination of training to new staff in the busy ED staff;

‘*There is a study website and there’s an investigator-only section that you can log onto, and then there’s training resources on there, including the videos….when they’ve got 5 minutes, just to sit and watch one of those videos*’ (P1, focus group 1, male, physician).

### Staff views on recruitment, training and trial conduct prior to the final stages of the trial

In the online questionnaire 8 months before the end of the trial, practitioners involved in any element of EcLiPSE were asked to select the statements (see Table 6) which they felt were relevant to their site. The majority indicated that the trial was running well, which supported trial recruitment data (recruiting to target with a 95% consent rate). Only two practitioners (2/199, 1%) reported that anxieties about RWPC were a barrier to recruitment. The majority indicated that their site held regular (e.g. monthly) EcLiPSE training sessions (134/199, 67%) for new staff or as refresher training. Practitioners did not feel that they would benefit from additional training (132/199, 66%). The sample included 75 (38%) practitioners who had not attended the initial SIV training. Some reported that staff shortages had led to patients being missed, whilst some (6%) indicated that training was not frequent enough.

**Table 6:** Staff views on trial conduct and training dissemination 8 months before anticipated trial end date (*n* = 199)

Statement	*n* (%)
The trial is running well and we have no issues	125 (63)
The trial is running well but we do have some issues	40 (20)
The trial is not running well as we have some issues	5 (3)
Staff shortages have led to patients being missed	13 (7)
There is a lack of support from the central EcLiPSE trial team	1 (1)
Site training is not frequent enough	11 (6)
It is difficult to find staff to cover consent seeking	5 (3)
There is a lack of support for EcLiPSE at site	2 (1)
Anxieties about research without consent are a barrier to recruitment	2 (1)

### Limitations

The study has some limitations. Although the majority of sites (87%) took part in the evaluation, only 47% of eligible practitioners anticipated staying for the full consent training and completed the questionnaires. Of these, 16% were excluded from analysis due to incomplete questionnaires. This attrition was because practitioners had to leave training early due to clinical commitments. This limitation reflects the challenge of delivering SIV training alongside ED clinical care commitments and highlights the importance of an effective, ongoing training dissemination strategy to ensure that all staff are able to access full training after a SIV. We were unable to re-administer the SIV questionnaire to the same participants at a later time point due to staff turnover. However, our study was strengthened by the conduct of interviews, focus groups and an online questionnaire throughout the trial with staff who had and had not attended the SIV. This mixed-method approach provided insight into multiple perspectives to assist understanding of the longer term uptake and impact of training upon practitioner confidence in recruitment and RWPC [16, 25] and potential barriers to trial success. Findings from the Consent study were used to inform support and feedback from the trial team to sites as part of an iterative approach. Finally, focus groups were conducted up to 18 months after SIV training due to the delays at sites that had been slow to open or recruit patients. This may have impacted upon practitioner recall about how they felt before SIV training and their views may have been influenced by trial recruitment experience.

## Discussion

Our findings demonstrate how a 4-h interactive site training meeting can significantly alleviate practitioner concerns about recruitment and consent in a challenging trial in paediatric emergency medicine. Successful patient recruitment to EcLiPSE is dependent upon practitioners in many different departments, specifically the ED, general paediatrics, paediatric neurology and paediatric intensive care, being aware of, and comfortable with, the trial protocol. For many, this was the first time that teams would be working together to ensure that critically ill children were screened and randomised, whilst ensuring that families were appropriately approached to discuss the trial after the time-critical emergency situation had passed. Consequently, it was not surprising that before site training many practitioners had concerns about conducting EcLiPSE and lacked confidence in how to communicate some elements of the trial, including RWPC, to families.

Practitioners rated highly the clarity and content of SIV training. Significant improvements were observed in practitioners’ confidence in explaining the study, and randomisation, and RWPC to families, as well as how to respond to parents who might object to their child being randomised during an emergency resuscitation. A previous survey published by members of our group [10] showed how practitioners with previous experience of RWPC in a medical device trial (the CATCH trial) [26] had more positive perceptions of this method when compared to those without such experience. However, our questionnaire data show that before training, previous experience of RWPC was not associated with significantly reduced concerns or greater confidence in recruitment and consent in EcLiPSE. This may reflect the challenging nature of the trial, in that it was a CTIMP, involving a change in usual ED clinical practice and, for many, their first ED-led clinical trial.

Site training provides an opportunity to discuss and learn about the potential challenges and solutions to trial recruitment and conduct [27, 28]. Our findings suggest that the use of training videos complemented this process and helped practitioners to visualise potentially difficult trial processes, including screening in a resuscitation situation. Practitioners particularly valued the RWPC video, which had been informed by data on parents’ views and priorities for trial information from pre-trial research [11] and CONNECT study guidance on RWPC [7, 13]. The video provided practitioners with examples of how to communicate RWPC to parents [7, 13], as well as preparing them for the sorts of questions that parents might ask about the trial [11].

The involvement and commitment of the whole team is required in educational activities to facilitate successful trial recruitment [27]. Nevertheless, logistical concerns about having adequate numbers of staff to support the trial across departments could not be fully addressed through training. These concerns are currently relevant to all research conducted within the UK National Health Service; it is particularly pertinent to research led by EDs working under increasing pressures. Importantly, despite concerns over challenges in trial delivery, at the time of writing, EcLiPSE had completed recruited within the expected timeframe and achieving the recruitment target. Consent was provided for 385 of 404 (95.3%) randomised participants.

Although practitioner confidence in recruitment and consent and trial success are clearly important outcomes, arguably it is also important to establish whether EcLiPSE training improved the quality of consent discussions, and parental experiences of recruitment and consent in EcLiPSE. These questions will be explored as part of the ongoing Consent study [6].

## Conclusion

Interactive SIV training can improve practitioners’ confidence in conducting research in a time-critical paediatric trial, which involves randomisation of children without prior informed consent. Our findings highlight the value of using parents’ perspectives to inform training and to engage practitioners in trials that are at high risk of being too challenging to conduct.

## Additional files

Additional file 1: Site initiation visit (SIV) questionnaire. (DOCX 647 kb)

Additional file 2: Example telephone interviews and focus group topic guide questions related to site visit training. (DOCX 18 kb)
