# Supplementary material for: Enhancing practitioners’ confidence in recruitment and consent in the EcLiPSE trial: a mixed-method evaluation of site training – a Paediatric Emergency Research in the United Kingdom and Ireland (PERUKI) study
Source: Trials. 2019 Mar 21;20:181. doi: 10.1186/s13063-019-3273-z (PMC6429745; doi:10.1186/s13063-019-3273-z)
Supplement: Supplementary file 1 — Site initiation visit (SIV) questionnaire. (DOCX 647 kb) [file 13063_2019_3273_MOESM1_ESM.docx]

**Additional file 1: SIV questionnaire**

****Part A- PLEASE COMPLETE IN ADVANCE OF EcLiPSE Site Initiation Visit ****

**“Emergency Treatment with Levetiracetam or Phenytoin in Status Epilepticus in Children (EcLiPSE) – an open label randomized controlled trial”**

We are keen to explore your views and experiences of the EcLiPSE Site Initiation Visit to help us develop the training we provide. Please complete the questions below, which should take no more than 5 minutes. Please complete Part A of the questionnaire before the session begins. Please complete PART B at the end of the session. Your answers will be kept confidential. Your views will help us with other EcLiPSE SIV’s.

Gender (please circle): *Male/Female*

Experience of recruitment to clinical research: *months / years* (please circle)

Role (please circle):

ED Nurse ED Clinician Research Nurse Ward Nurse/ Doctor

Other (Please state)

--------------------------------------------------------------------------------------------------------------------------

1. Do you have any previous experience of seeking deferred consent? Yes/ No

a. If no, have you had any other involvement in deferred consent? (Please elaborate)

b. If yes please state the name of the trial/s:

2. Please rate these statements 1-5 with 1= strongly disagree, 2=mildly disagree, 3= neutral, 4= mildly agree, 5= strongly agree

a. I feel confident in explaining the study to families

b. I feel confident when explaining randomization to families

c. I feel confident in explaining deferred consent to families

d. I feel confident in dealing with parents who object to their child being randomized

3. Do you have any concerns about recruiting to EcLiPSE? Yes /No

3a. Please elaborate:

4. Do you have any concerns about seeking deferred consent for EcLiPSE? Yes/No

4a. Please elaborate:

5. Do you think there will be any practical or logistical difficulties with conducting EcLiPSE? Yes/No 5a. Please elaborate:

6. If there is anything else you want to mention about this trial, please do so in the space below.

****Part B- PLEASE COMPLETE AFTER EcLiPSE Site Initiation Visit****

**“Emergency Treatment with Levetiracetam or Phenytoin in Status Epilepticus in Children (EcLiPSE) – an open label randomized controlled trial”**

Could you please complete the questions below and return to the study team before you leave today? Your answers will be kept confidential.

**Were you part of the group of people who have received the ‘full’ EcLiPSE training today, including the deferred consent training by Kerry or Louise delivered separately to the ED training section? Yes / No**

1. Please rate these statements 1-5 with 1= strongly disagree, 2=mildly disagree, 3= neutral, 4= mildly agree, 5= strongly agree

a. I feel confident in explaining the study to families

b. I feel confident when explaining randomization to families

c. I feel confident in explaining deferred consent to families

d. I feel confident in dealing with parents who object to their child being randomized


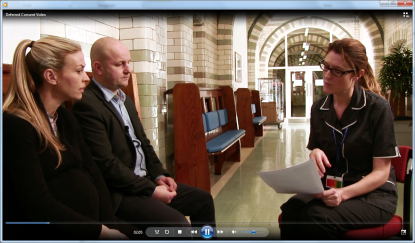
e. The consent training video helped me feel more confident about seeking

deferred consent. Please elaborate:

f. The screening video helped me feel more confident about identifying eligible children


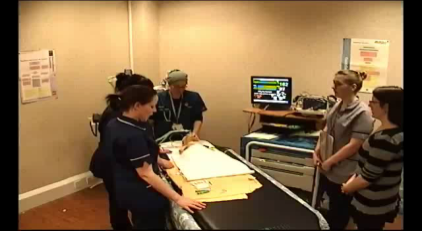
Please elaborate:


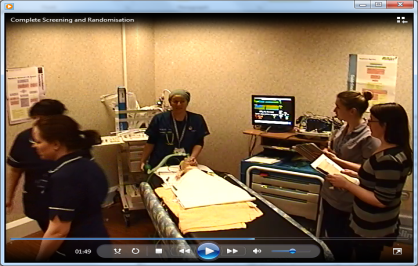
g. The randomization video helped me feel more confident about how to randomize

children to the trial and complete the screening log

Please elaborate:

2. Do you have any concerns or anxieties specific to this trial, including the deferred consent approach we have discussed? Please elaborate:

3. Would you like to be supported further during this trial? Yes/ No

3a. If so, please tell us how you would like to be supported

(Examples could include further face to face training on specific issues, emails/post/website/ updates from the EcLiPSE team, podcasts with training tips, or anything else you can think of)

4. Looking around the room, do you think that everyone who needs to be at this SIV is here? Yes/No

4a. If not, could you please tell us who we will need to include to make this trial a success?

(Please mention job roles or individuals)

5. Please could you rate the content and clarity of the slides and information using a scale of 1-5 with 1=Poor, 2=Not very good, 3=Satisfactory, 4=Good, 5=Excellent

|  | Content | Clarity |
| --- | --- | --- |
| a. Introduction to EcLiPSE |  |  |
| b. Protocol Overview |  |  |
| c. Deferred Consent |  |  |
| d. Safety reporting & monitoring |  |  |
| e. The ED essentials |  |  |

6. If there is anything else you would like to share with us about this trial or anything that would help to make the trial a success please let us know in the space below.
