# Supplementary material for: Enhancing practitioners’ confidence in recruitment and consent in the EcLiPSE trial: a mixed-method evaluation of site training – a Paediatric Emergency Research in the United Kingdom and Ireland (PERUKI) study
Source: Trials. 2019 Mar 21;20:181. doi: 10.1186/s13063-019-3273-z (PMC6429745; doi:10.1186/s13063-019-3273-z)
Supplement: Supplementary file 2 — Example telephone interviews and focus group topic guide questions related to site visit training. (DOCX 18 kb) [file 13063_2019_3273_MOESM2_ESM.docx]

**Additional file 2: Example telephone interviews and focus group topic guide questions related to site visit training**

| Interview topics | Example questions |
| --- | --- |
| Knowledge and experience pre- training | *- Please define your role in EcLiPSE*  *- Prior to EcLiPSE did you have any previous experiences of trial recruitment?* If yes, long have you worked in trials? *Have you been in any trials that used research without prior consent (deferred consent)?*  *- How many children have you personally recruited to EcLiPSE?*  *- How many children have your site recruited to EcLiPSE?* |
| Thoughts about EcLiPSE and practitioner engagement with the trial | *- What were your thoughts about EcLiPSE before the SIV? Prompt: did you have any concerns?*  *- What was the feeling amongst your site colleagues about EcLiPSE before the meeting?*  *- Do you think there was support for the trial? Prompt: was there ‘buy in’ for this study before the meeting?*  *- What do you think helps with getting ‘buy in’ from the site team?*  *- What were your thoughts about EcLiPSE after the SIV? Prompt: did you have any concerns?* |
| Experience and of training | *- Is there anything that stands out about the training? Prompt: anything you found particularly useful/anything not useful*  *- What did you think about the randomization video? Prompts: How useful was it? Anything you didn’t find useful? Did you watch it again after the training?*  *- What did you think about the consent video? Prompts: How useful was it? Anything you didn’t find useful? Did you watch it again after the training?*  *- Did you have a simulation? Prompts: How useful was it? How could it have been improved?* |
| Improving the training visit | *- Do you have any comments on how the training could work better? Prompt: explore views on;*   - *The invitations and expectations of the training* - *The length of the training* - *The members of the EcLiPSE team present* - *The members of the site staff present* - *What worked well in the training* |
| Dissemination of training at site | *- Who is repeating/ disseminating the training since the SIV visit or who plans to?*  *- How is the dissemination of training going?*  *- Are there groups of staff who are particularly difficult to reach in disseminating the training?*  *- How many times has the training been repeated?*  *- How regular is the training/ updates on EcLiPSE? Does this differ for new staff and existing staff?* |
| Consent process experience | *- How have parents reacted to finding out that their child has been entered into a clinical trial without their prior consent?*  *- Have your views about research without prior consent changed over time?* |
